# Public health round-up

**DOI:** 10.2471/BLT.18.010118

**Published:** 2018-01-01

**UN leaders call for full lifting of Yemen sea blockade**

United Nations leaders called on the Saudi-led coalition to fully lift the blockade of Yemeni Red Sea ports to allow commercial imports of food, fuel and medicines. On 2 December 2017, seven heads of United Nations agencies said that a partial lifting of the blockade in November to allow humanitarian deliveries would not prevent “a massive humanitarian tragedy costing millions of lives” in Yemen. This photo shows a diarrhoea treatment centre in the capital Sana’a.

http://bit.ly/2BzNZbH

**Figure:**
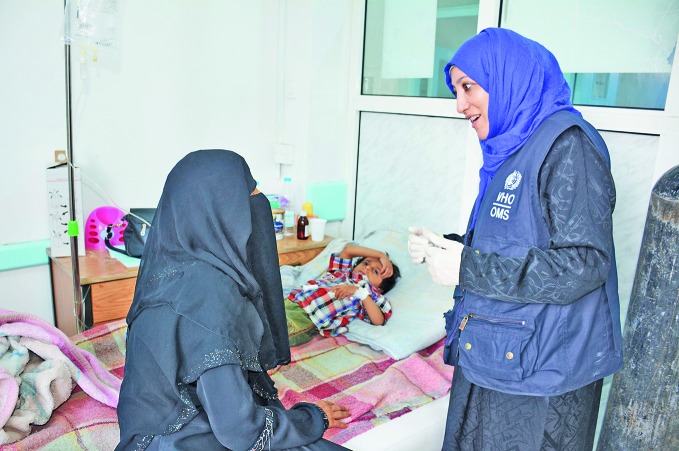
WHO

## Malaria progress stalled

After an unprecedented period of success in global malaria control, progress has stalled in recent years due mainly to insufficient investment in malaria programmes, according to the *World malaria report 2017* released last month.

In 2016, there were an estimated 216 million cases of malaria in 91 countries, an increase of about 5 million over 2015. Deaths reached 445 000 in 2016, a similar estimate to the previous year, according to the World Health Organization (WHO) report.

While the incidence of new cases of malaria has fallen overall, the trend since 2014 has levelled off and even reversed in some regions. Malaria mortality followed a similar pattern, the report said.

“We are now at a turning point. Without urgent action, we risk going backwards, and missing the global malaria targets for 2020 and beyond,” said Dr Tedros Adhanom Ghebreyesus, Director-General of WHO.

The WHO *Global technical strategy for Malaria* calls for reductions of at least 40% in malaria case incidence and mortality rates by the year 2020. According to the report, the world is not on track to meet these goals.

A major problem is insufficient funding at both domestic and international levels, resulting in gaps in coverage of insecticide-treated nets, medicines, and other life-saving tools. 

An estimated US$ 2.7 billion was invested in malaria control and elimination efforts globally in 2016, far short of the US $6.5 billion annual investment needed by 2020 to meet the 2030 targets of the WHO global malaria strategy.

In 2016, governments of endemic countries provided US$ 800 million, representing 31% of total funding.

http://bit.ly/2inCEUM

## Europe’s HIV epidemic

WHO’s European Region is the only region worldwide where the number of new HIV infections is rising, HIV surveillance data from 2016 showed.

More than 160 000 people were newly diagnosed across the 53-country region, according to a joint HIV surveillance report by the WHO Regional Office for Europe and European Centre for Disease Prevention and Control released ahead of World AIDS Day on 1 December 2017.

More than half (51%) of the new HIV cases reported in 2016 indicated that they were detected at a late stage of infection.

“The HIV epidemic continues to rise at an alarming pace in the European Region, mostly in its eastern part, which is home to almost 80% of the 160 000 new HIV diagnoses,” said Dr Zsuzsanna Jakab, WHO Regional Director for Europe.

“This is the highest number of new cases ever recorded in one year [in the WHO European region]. If this trend persists, we will not be able to achieve the sustainable development goal (SDG) target of ending the HIV epidemic by 2030,” she said.

WHO is working with countries in the region to update their HIV counselling and testing policies and to adapt effective HIV testing practices using WHO’s consolidated guidelines.

http://bit.ly/2zO1Xq0

## Substandard and falsified medicines

WHO Director-General Dr Tedros Adhanom Ghebreyesus called on countries to step up their efforts to tackle the problem of substandard and falsified medicines, since quality medicines are essential for achieving universal health coverage.

An estimated 1 in 10 medical (10%) products in low- and middle-income countries is either substandard or falsified, according to a new WHO study.

The study, *Public health and socioeconomic impact of substandard and falsified medical products*, published on 28 November 2017, reviewed more than 100 articles on medicine quality surveys in 88 low- and middle-income countries involving 48 000 samples.

Based on the 10% estimate, a modelling exercise by the University of Edinburgh in Scotland calculated that between 72 000 and 169 000 children may be dying each year from pneumonia due to substandard and falsified antibiotics.

A second study by researchers from the London School of Hygiene and Tropical Medicine estimated that substandard and falsified medicines, could be a factor in 116 000 malaria deaths in sub-Saharan Africa.

Substandard and falsified medicines particularly affect the most vulnerable communities that struggle to access essential medicines. They often occur due to low access, weak technical capacity and poor governance, including corruption.

To coincide with the release of the studies, WHO published a compilation of about 1500 case reports of substandard and falsified medical products it has received since 2013, when the WHO Global Surveillance and Monitoring System was established.

These case reports are from all six WHO regions and cover a wide range of products from cancer treatments to contraception, both branded and generic. The most commonly reported reports were of substandard or falsified antimalarials and antibiotics.

WHO has trained 550 regulators from 141 countries over the last four years to detect and control these products.

http://bit.ly/2iQ2Mv8

## Yellow fever in Nigeria

The International Coordinating Group on vaccine provision for yellow fever has provided 1.4 million vaccine doses for an immunization campaign that started on 2 December to help quell an outbreak in Nigeria.

The Nigerian government planned to vaccinate 1.3 million people in parts of Zamfara state in northwestern Nigeria, where cases of the mosquito-borne disease have been confirmed, with support from WHO and other partners.

A pre-emptive campaign to protect people in areas at high risk of yellow fever transmission is also being planned.

The first case of yellow fever was confirmed in August 2017. Since then, 276 suspected cases have been reported in 14 states and, in addition, 30 confirmed cases have been reported in four of those states: Kogi, Kwara, Kano and Zamfara.

Some 874 000 people were immunized against the acute viral haemorrhagic disease in Kwara and Kogi states with International Coordinating Group vaccines.

The International Coordination Group coordinates the timely and equitable provision of vaccines during outbreaks. It maintains an emergency stockpile of six million doses of yellow fever vaccine, which is continually replenished. It is funded by Gavi, the Vaccine Alliance.

http://bit.ly/2BA2Y5p

**Cover photo**

A five-year-old girl washes her hands in the Naryn province of Kyrgyzstan, as part of a daily routine using the outdoor basin complete with a mirror, step stool, soap, toothbrush and cupboard.

**Figure:**
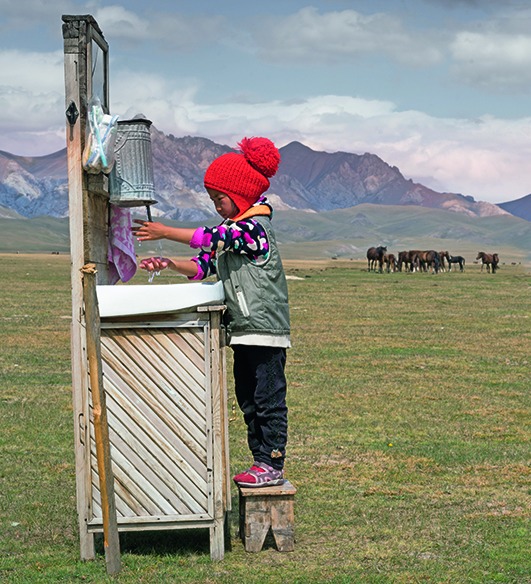
© UNICEF/ Simon Lister

## WHO Africa nutrition report

Undernutrition in children still persists and childhood obesity is a growing problem in WHO’s African Region, according to the *Africa nutrition report* released in November 2017.

The prevalence of stunting decreased between 2000 and 2016, but the absolute numbers of stunted children increased from an estimated 50.4 million in 2000 to 58.5 million in 2016. The stunting prevalence in the majority of countries exceeds 30% and is a serious public health concern.

The report describes the current status in relation to six global nutrition targets that WHO Member States have committed to achieve by 2025.

Many countries in the region still report wasting rates in children that are above the target of 5% or below, while only 17 countries in the region meet that target.

The estimated prevalence of wasting in a further 19 countries is 5–9%, in six countries it is 10–14% and in three countries it exceeds the 15% threshold that signals a public health emergency: Eritrea 15.3%, Niger 18.7% and South Sudan 22.7%.

Stunting, or impaired growth and development, tends to occur before a child reaches the age of two. Wasting, or low weight compared to the height of a child, is a strong predictor of mortality among children under five.

The report is based on data from national surveys of the 47 countries dating as far back as 2000, as well as joint malnutrition estimates published annually by the United Nations Children’s Fund (UNICEF), WHO and the World Bank.

With more than 890 million inhabitants in 47 countries, the WHO African Region accounts for about 12% of the world’s population.

The report also highlights gaps in the data. For 19 of the 47 countries in the region, the most recent data are from 2012. In two countries, the most recent surveys pre-date 2000.

Joint UNICEF, WHO and World Bank 2016 estimates show that the number of overweight children in Africa increased by more than 50% between 2000 and 2015.

http://bit.ly/2AWDrYa

## Guideline for radiological emergencies

A new WHO guideline recommends the provision of iodine to anyone at risk of being exposed to radioiodine, as an urgent protective measure before or during a radiological or nuclear emergency.

Inhalation of air or the consumption of food and drinking water contaminated with radioactive iodine during a nuclear accident may lead to internal radiation exposure and uptake of radioactive iodine mainly by the thyroid.

Iodine thyroid blocking should be provided within the frame of a justified and optimized protection strategy, according to *Iodine thyroid blocking: guidelines for use in planning and responding to radiological and nuclear emergencies*.

The new guideline can be used by emergency planners, policy–makers, public health specialists and clinicians to strengthen public health preparedness for radiation emergencies, as required by the International Health Regulations and in line with international safety standards (GSR Part 7).

The guideline is confined to the public health aspects of planning and implementation of iodine thyroid blocking in an emergency, such as dosage and timing of administration, adverse effects of stable iodine, its packaging, storage, and distribution. It supersedes 1999 WHO *Guidelines for iodine prophylaxis following nuclear accidents*.

http://bit.ly/2kkLMO5

**Looking ahead**

22–27 January – 142nd Executive Board meeting

4 February – World Cancer Day

21–26 May – World Health Assembly

